#  Condições de saúde autorrelatadas por solicitantes de refúgio no Rio de Janeiro, Brasil, de 2010 a 2017 

**DOI:** 10.1590/0102-311XPT068623

**Published:** 2023-09-18

**Authors:** Raquel Proença, João Roberto Cavalcante, Anete Trajman, Eduardo Faerstein

**Affiliations:** 1 Instituto de Medicina Social, Universidade do Estado do Rio de Janeiro, Rio de Janeiro, Brasil.; 2 Universidade Federal do Rio de Janeiro, Rio de Janeiro, Brasil.

**Keywords:** Refugiados, Perfil de Saúde, Migração Internacional, Saúde Global, Refugees, Health Profile, International Migration, Global Health, Refugiados, Perfil de Salud, Migración Internacional, Salud Global

## Abstract

No Brasil, entre 2011 e 2022, 348.067 pessoas solicitaram o reconhecimento da condição de refugiado no país. Os motivos que resultaram na migração, os riscos durante o trajeto e a transição cultural ao chegar podem estar associados a diferentes problemas de saúde. O objetivo deste estudo foi analisar as condições de saúde autorrelatadas por solicitantes de refúgio no Município do Rio de Janeiro no período de 2010 a 2017. Trata-se de um estudo transversal de dados secundários. Foram coletadas informações preenchidas nos formulários de solicitação de refúgio do Comitê Nacional para os Refugiados (Conare) de 2010 a 2017 e da entrevista social da Cáritas Arquidiocesana do Rio de Janeiro (Cáritas/RJ). Calcularam-se as taxas de prevalência de condições de saúde e respectivos intervalos de 95% de confiança (IC95%) e a razão de chances (RC) e IC95% em um modelo de regressão logística simples segundo variáveis sociodemográficas e de migração. O estudo incluiu 1.509 indivíduos. Na chegada ao Brasil, 620 (41%) relataram ter uma ou mais condições de saúde. As chances de apresentar problemas de saúde foram maiores em pessoas oriundas do Congo (RC = 18,7) e República Democrática do Congo (RC = 9,5), nos indocumentados (RC = 4,4), nas mulheres (RC = 2,1), em pessoas com Ensino Fundamental (RC = 1,9), com idade ≥ 45 anos (RC = 1,8) e entre os que vivem/viveram maritalmente (RC = 1,8 e 2,5, respectivamente). Entre as pessoas que relataram alguma condição de saúde, mais da metade informaram sentir dores (52%). É possível que as dores físicas tenham relação com estresse pós-traumático e outros sofrimentos em saúde mental, que podem se manifestar por meio de sintomas de dores somáticas.

## Abstract (en)

From 2011 to 2022, 348,067 people applied for a refugee status in Brazil. The reasons that resulted in their migration, the risks during their journey, and the cultural transition upon arrival may be associated with different health problems. This study aimed to analyze the self-reported health conditions of asylum seekers in the municipality of Rio de Janeiro from 2010 to 2017. This is a cross-sectional study with secondary data. Data were collected from asylum application forms at the Brazilian National Committee for Refugees (Conare) from 2010 to 2017 and from social interviews in the Archdiocesan Caritas of Rio de Janeiro (Cáritas/RJ). The prevalence rates of health conditions, their respective 95% confidence intervals (95%CI) and odds ratio (OR) were calculated in a simple logistic regression model according to sociodemographic and migration variables. This study included 1,509 individuals. Upon arrival in Brazil, 620 (41%) reported having one or more health conditions. The chances of showing health problems were higher in people from the Congo (OR = 18.7) and the Democratic Republic of the Congo (OR = 9.5), in undocumented individuals (OR = 4.4), women (OR = 2.1), in people with elementary education (OR = 1.9), aged ≥ 45 years (OR = 1.8), and among those who live/lived maritally (OR = 1.8 and 2.5, respectively). Of those who reported a health condition, more than half claimed experiencing pain (52%). Physical pain may be related to post-traumatic stress and other mental health distress, manifesting itself by somatic pain symptoms.

## Abstract (es)

En Brasil, entre el 2011 y el 2022, 348.067 personas solicitaron el reconocimiento de la condición de refugiado en el país. Los motivos que dieron lugar a la migración, los riesgos durante el trayecto y la transición cultural al llegar pueden estar asociados a diferentes problemas de salud. Este estudio tuvo como objetivo analizar las condiciones de salud autoinformadas de los solicitantes de refugio en el municipio de Río de Janeiro en el período del 2010 al 2017. Se trata de un estudio transversal de datos secundarios. Los datos se recopilaron de los formularios de solicitud de asilo del Comité Nacional para Refugiados de Brasil (Conare) del 2010 al 2017 y de la entrevista social de Caritas Arquidiocesana de Río de Janeiro (Cáritas/RJ). Se calcularon las tasas de prevalencia de las condiciones de salud y sus respectivos intervalos de 95% de confianza (IC95%) y la razón de posibilidades (RP) y el IC95% en un modelo de regresión logística simple según variables sociodemográficas y de migración. El estudio incluyó a 1.509 sujetos. A su llegada a Brasil, 620 (41%) informaron tener una o más condiciones de salud. Las posibilidades de presentar problemas de salud fueron mayores en personas procedentes de Congo (RP = 18,7) y República Democrática del Congo (RP = 9,5), en personas indocumentadas (RP = 4,4), mujeres (RP = 2,1), en personas con educación primaria (RP = 1,9), en ≥ 45 años (RP = 1,8), y entre los que viven/han vivido en una relación estable (RP = 1,8 y 2,5, respectivamente). Entre las personas que reportaron alguna condición de salud, más de la mitad relató sentir dolores (52%). Los dolores físicos pueden estar relacionados con el estrés postraumático y otros sufrimientos en salud mental que pueden presentarse mediante síntomas de dolores somáticos.

## Introdução

A Convenção das Nações Unidas sobre Refugiados de 1951 define refugiado como alguém que “*temendo ser perseguido por motivos de raça, religião, nacionalidade, grupo social ou opiniões políticas, se encontra fora do país de sua nacionalidade e que não pode ou, em virtude desse temor, não quer valer-se da proteção desse país*” ^1^. O Alto Comissariado das Nações Unidas para Refugiados (ACNUR) informou que em 2022 havia aproximadamente 108,8 milhões de migrantes forçados no mundo ^2^.

Entre 2011 e 2022, 348.067 pessoas solicitaram o reconhecimento da condição de refugiado no Brasil, das quais 50.355 (14,5%) requisitaram em 2022 ^3^. O local de nascimento dos solicitantes de refúgio no Brasil mudou ao longo da última década, passando de países africanos, majoritariamente, devido a guerras civis e conflitos internos, para países sul-americanos, em razão de desastres naturais, epidemias e violação de direitos humanos ^1^^,^^2^. A migração forçada em massa de venezuelanos para o Brasil ocorreu mais recentemente, a partir de 2016, causada pela grave crise humanitária no país ^3^. Das 50.355 solicitações de refúgio realizadas em 2022 no Brasil, 33.753 (67%) foram provenientes de venezuelanos ^3^.

A migração forçada pode impactar a saúde de solicitantes de refúgio antes, durante ou depois do deslocamento migratório ^4^^,^^5^^,^^6^^,^^7^^,^^8^. Entre outras, são descritas lesões físicas, dores, diarreia, infecções respiratórias - incluindo tuberculose -, depressão e ansiedade, estresse pós-traumático, doenças crônicas não transmissíveis, fome e desnutrição ^4^^,^^5^^,^^6^^,^^7^^,^^8^. Ainda que solicitantes de refúgio e refugiados estejam sujeitos aos mesmos determinantes sociais em saúde que a população nacional, há singularidades nesses casos, como violências e xenofobia sofridas durante e depois do processo migratório e dificuldade de acesso aos serviços de saúde no país de destino ^8^^,^^9^.

Poucas pesquisas brasileiras descreveram o perfil de saúde de solicitantes de refúgio ou refugiados ^9^^,^^10^. O objetivo deste estudo foi analisar as condições de saúde autorrelatadas por solicitantes de refúgio no Município do Rio de Janeiro no período de 2010 a 2017.

## Métodos

Este é um estudo transversal de dados secundários feito com solicitantes de refúgio que informaram sobre suas condições de saúde em pelo menos uma das fontes de dados. As informações foram extraídas do formulário de solicitação de refúgio do Comitê Nacional para os Refugiados (Conare) e da entrevista social da Cáritas Arquidiocesana do Rio de Janeiro (Cáritas/RJ) entre 2010 e 2017. O formulário era disponibilizado para os solicitantes de refúgio em português, espanhol, inglês ou francês e, quando necessário, era fornecida a ajuda de um intérprete. Após 2017, os questionários sofreram modificações substanciais, e as perguntas sobre saúde foram retiradas do formulário do Conare.

Foram realizadas três etapas de pré-testes da máscara de inserção de dados criada no software EpiData, versão 4.2.0.0 (http://www.epidata.dk/), para os formulários do Conare. Durante o período de coleta (abril de 2018 a dezembro de 2019), semanalmente, os formulários coletados pelos digitadores eram verificados e corrigidos. Foram encontradas 254 divergências entre todos os formulários, considerando que cada um contém em média 148 variáveis, os erros de digitação representaram 0,6%. Nenhum formulário deixou de ser incluído no estudo por má conservação da ficha.

O número de identificação do solicitante ou núcleo familiar do formulário e a data de nascimento foram utilizados como critérios de agrupamento para vincular os dados nas duas fontes. Nos casos de divergência de datas, o formulário foi excluído (possível preenchimento por diferentes familiares).

As prevalências de condições de saúde e respectivos intervalos de 95% de confiança (IC95%) foram calculados ^11^. Exploramos a associação das variáveis sociodemográficas e de migração com o desfecho (presença de condições de saúde), via razão de chances (RC) e IC95%, em um modelo de regressão logística simples. Essas variáveis incluíram país de nascimento, sexo, faixa etária, escolaridade, estado civil, meios de transporte na migração, *status* de documentação e *status* de refúgio.

O estudo foi aprovado pelo Comitê de Ética em Pesquisa do Instituto de Medicina Social Hesio Cordeiro da Universidade do Estado do Rio de Janeiro (parecer nº 2.437.258, de 14 de dezembro de 2017).

## Resultados

Entre os 2.287 solicitantes de refúgio atendidos na Cáritas/RJ entre 2010 e 2017, foram incluídos 1.509 indivíduos, 719 foram excluídos por não preencherem uma das fontes de dados, 22 por não atenderem aos critérios de agrupamento e 37 por não haver, em nenhuma das fontes, informação sobre condições de saúde. A maior parte era do sexo masculino (976; 64,7%), solteiro (974; 64,5%), tinha entre 25 e 44 anos (1.072; 71%) e chegou ao Brasil por meio de transporte aéreo (1.234; 81,8%) (Tabela 1).

**Tabela 1:** Prevalências e razão de chances (RC) de condições de saúde autorrelatadas e respectivos intervalos de 95% de confiança (IC95%) segundo características sociodemográficas de solicitantes de refúgio atendidos na Cáritas Arquidiocesana do Rio de Janeiro (Cáritas/RJ), 2010 a 2017.

Características	n	%	Prevalência * (IC95%)	RC (IC95%)
Geral	1.509	100,0	41 (38-43)	-
Sexo				
Masculino	976	64,7	34 (31-37)	1,0 (Referência)
Feminino	531	35,2	53 (49-57)	2,1 (1,8-2,7)
Não informado	2	0,1	-	-
Faixa etária (anos)				
15-24	341	22,6	37 (32-42)	1,0 (Referência)
25-44	1.072	71,0	41 (38-44)	1,2 (0,9-1,5)
45 ou mais	95	6,3	52 (41-62)	1,8 (1,1-2,8)
Não informado	1	0,1	-	-
Estado civil				
Solteiro	974	64,5	36 (33-39)	1,0 (Referência)
Vive junto	435	28,8	49 (45-54)	1,8 (1,4-2,2)
Já viveu junto	63	4,2	59 (47-71)	2,5 (1,5-4,3)
Não informado	37	2,5	-	-
Escolaridade				
Superior	523	34,7	35 (31-39)	1,0 (Referência)
Fundamental	267	17,7	51 (45-57)	1,9 (1,4-2,6)
Médio	629	41,7	42 (38-46)	1,3 (1,0-1,7)
Não informado	90	6,0	-	-
Meio de transporte utilizado				
Aéreo	1.234	81,8	39 (37-42)	1,0 (Referência)
Marítimo e terrestre	216	14,3	47 (40-53)	1,3 (1,0-1,8)
Não informado	59	3,9	-	-
*Status* da solicitação de refúgio				
Pendente	560	37,1	39 (35-43)	1,0 (Referência)
Aceito	237	15,7	43 (37-50)	3,7 (1,7-9,5)
Recusado	41	2,7	17 (5-29)	3,1 (1,4-7,7)
Não informado	671	44,5	-	-
*Status* da documentação				
Documentado	985	65,3	31 (28-34)	1,0 (Referência)
Indocumentado	87	5,8	67 (57-78)	4,4 (2,8-7,1)
Documento falso ou emprestado	18	1,2	44 (21-67)	1,8 (0,7-4,5)
Não informado	419	27,8		
País de nascimento				
Síria	42	2,8	14 (4-25)	1,0 (Referência)
Congo	37	2,5	76 (62-90)	18,7 (6,3-63,7)
República Democrática do Congo	544	36,1	61 (57-65)	9,5 (4,2-25,3)
Angola	251	16,6	37 (31-43)	3,5 (1,5-9,6)
Guiné-Bissau	70	4,6	29 (18-39)	2,4 (0,9-7,1)
Nigéria	24	1,6	29 (11-47)	2,3 (0,7-8,8)
Colômbia	113	7,5	27 (19-36)	2,3 (0,9-6,4)
Senegal	41	2,7	27 (13-40)	2,2 (0,7-7,0)
Venezuela	94	6,2	21 (13-29)	1,6 (0,6-4,7)
Guiné	23	1,5	17 (2-32)	1,3 (0,3-5,0)
Outros	261	17,3	-	-
Não informado	9	0,6	-	-

Nota: alguns indivíduos não especificaram o tipo de condição de saúde relatada. Um indivíduo podia relatar mais de uma condição de saúde.

* Prevalência por 100 pessoas (não informado incluído no cálculo do denominador).

**Figura 1:**
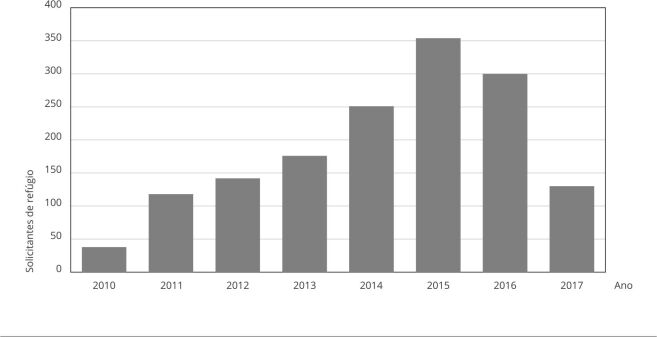
Distribuição dos indivíduos segundo ano de preenchimento do formulário dos solicitantes de refúgio atendidos na Cáritas Arquidiocesana do Rio de Janeiro (Cáritas/RJ), 2010 a 2017 (n = 1.509).

Relataram ter uma ou mais condições de saúde 620 (41%) indivíduos; os demais 889 não mencionaram nenhuma condição de saúde. As chances de relatar problemas de saúde foram maiores entre oriundos do Congo (RC = 18,7) e República Democrática do Congo (RC = 9,5), indocumentados (RC = 4,4), mulheres (RC = 2,1), pessoas com Ensino Fundamental (RC = 1,9), com idade ≥ 45 anos (RC = 1,8) e entre os que vivem/viveram maritalmente (RC = 1,8 e 2,5, respectivamente) (Tabela 1). A maior parte de solicitações de refúgio (905; 60% da amostra) se deu nos anos 2014, 2015 e 2016 (Figura 1).

Foram autorrelatadas 877 condições de saúde (Tabela 2); mais da metade informaram sentir dores (320; 51,6%), dentre os quais 6 (2%) relataram que a dor era resultado de tortura ou violência física sofridas.

**Tabela 2:** Classificação das condições de saúde autorrelatadas por solicitantes de refúgio atendidos na Cáritas Arquidiocesana do Rio de Janeiro (Cáritas/RJ), 2010 a 2017 (n = 620).

Condições de saúde autorrelatadas	n	%	IC95%
Número de condições de saúde relatadas por uma mesma pessoa			
1	404	65,2	61,4-69,0
2	142	22,9	20,0-26,2
3	44	7,1	5,1-9,1
4	13	2,1	1,0-3,2
5	1	0,2	0,1-0,4
Não informado	16	2,6	-
Classificação das condições de saúde			
Dores	320	51,6	47,7-55,5
Oftalmológicos/Visão	73	11,8	9,2-14,3
Gastrointestinal	61	9,8	7,4-12,1
Gestante	51	18,0 *	13,5-22,4
Doenças crônicas não transmissíveis	41	6,6	4,7-8,5
Respiratórios	39	6,3	4,4-8,2
Saúde mental/Hábitos	39	6,3	4,4-8,2
Osteoarticular	37	6,0	4,1-7,8
Ginecológicos/Obstétricos	33	11,6 *	7,9-15,3
Doenças infecciosas	26	4,2	2,6-5,8
Dermatológico	23	3,7	2,2-5,2
Odontológicos	19	3,1	1,7-4,4
DCNT - cardíacas	17	2,7	1,4-4,0
Condições cirúrgicas	11	1,8	0,7-2,8
Alergia	10	1,6	0,6-2,6
Ouvido/Audição	8	1,3	0,4-2,2
Lesões	7	1,1	0,3-2,0
Urinário	6	1,0	0,2-1,7
Nutricionais	5	0,8	0,1-1,5
Outros sintomas	43	6,9	4,9-8,9
Outros	8	1,3	0,4-2,2

DCNT: doenças crônicas não transmissíveis; IC95%: intervalo de 95% de confiança.

Nota: alguns indivíduos não especificaram o tipo de condição de saúde. Um indivíduo podia relatar mais de uma condição de saúde.

* n = 284.

Das 51 gestantes (18% das mulheres), apenas uma estava realizando o acompanhamento pré-natal. Vinte e nove (4,7%) solicitantes de refúgio informaram que suas condições de saúde eram consequências de violências que sofreram, sendo que 6 eram pessoas vivendo com HIV/aids e duas eram gestantes; outras 7 atribuíram seus problemas de saúde às condições de fuga do país de origem.

Oito (1,3%) pessoas avaliaram que necessitam de tratamento de saúde e 9 (1,4%) informaram já estarem em tratamento no Brasil. Duas mulheres (0,7% entre as mulheres) afirmaram que precisam de tratamento ginecológico como consequência de violência sexual.

## Discussão

Neste estudo, encontramos alta prevalência (41%) de pelo menos uma condição de saúde autorrelatada entre os solicitantes de refúgio que procuraram a Cáritas/RJ. A prevalência foi maior nos grupos mais vulneráveis: mulheres, pessoas mais velhas, com menor grau de instrução, indocumentadas e oriundas de países onde há mais conflitos.

É difícil comparar essas prevalências com as da população brasileira em geral. No Brasil, os estudos que abordam morbidades autorrelatadas apresentam prevalências heterogêneas e foram realizados em subpopulações específicas, sujeitos, portanto, a diferentes vieses ^12^^,^^13^^,^^14^^,^^15^. No Brasil, pesquisas sobre autopercepção de saúde, como as da *Pesquisa Nacional de Saúde* (PNS), sugerem que, de forma geral, mulheres e idosos têm autopercepção de saúde melhor que homens e jovens ^16^. Solicitantes de refúgio e refugiados tendem a perceber suas condições de saúde melhor que a população nacional, pois o processo de migração está intrinsecamente ligado ao processo saúde e doença e à desassistência vivenciada no período pós-migração ^17^.

Os relatos sobre condições de saúde também são heterogêneos, pois geralmente os solicitantes de refúgio e refugiados têm necessidades de saúde específicas que, por vezes, podem refletir as doenças prevalentes nos países de origem ^17^. Os países cujas populações nacionais são mais abertas para receber solicitantes de refúgio e refugiados e que têm migrações ordenadas e seguras são Itália, Argentina, Holanda e Brasil, respectivamente, contudo, os países que mais recebem migrantes forçados no mundo hoje são Turquia, Irã, Colômbia e Alemanha ^18^.

Chamam a atenção a elevada proporção de gestantes e a insuficiente utilização dos serviços de saúde. Não sabemos se as gestantes já estavam cientes da gravidez ao migrarem ou se a gestação contribuiu para a decisão de migrar. É também digna de nota a frequência da dor como sintoma. Tem sido debatido o significado desse sintoma em pessoas vivendo em situação de refúgio ^19^. A dor relatada pode ser física ou mental e pode estar associada a estresse pós-traumático ou depressão ^20^^,^^21^.

Sofrimentos em saúde mental têm sido relatados em diversos estudos ^22^^,^^23^, mas, curiosamente, são pouco frequentes em nossa pesquisa, o que talvez se deva ao receio dos participantes de terem a solicitação de refúgio negada caso informem sofrimentos em saúde mental. Outra possível explicação seria a interpretação de sintomas, como tristeza, desânimo ou fadiga, que podem não ser reconhecidos como sofrimento em saúde mental ^24^. Perspectivas culturais distintas sobre origem de sofrimentos e aflições também podem influenciar o relato sobre sofrimentos em saúde mental ^25^.

Este estudo tem limitações. Os instrumentos de obtenção dos dados, formulário do Conare e entrevista social, não foram desenvolvidos para fins de pesquisa. O viés de informação, por receio de recusa de refúgio, é provável. A entrevista social era preenchida somente por uma pessoa do grupo familiar, portanto, algumas condições de saúde relatadas por outros familiares não foram incluídas nas análises. Estudos que utilizam dados de morbidade referida têm como objetivo conhecer a condição de saúde por meio do relato do próprio indivíduo, não sendo expressão direta de um diagnóstico médico.

Esta pesquisa analisou dados inéditos que podem ser importantes para a formulação de políticas públicas e de organizações não governamentais (ONG), como a Cáritas/RJ, que prestem serviços a essa população. O Sistema Único de Saúde (SUS) deve construir políticas de saúde que foquem nas necessidades de saúde dessa população que vive em vulnerabilidade, principalmente no âmbito da atenção primária em saúde, incluindo a assistência social e a atenção psicossocial.
